# Congenital Tracheal Stenosis Patients Undergoing Modified Slide Tracheoplasty: Single-Centre Technique and Long-Term Morbidity and Mortality

**DOI:** 10.1016/j.cjcpc.2023.10.003

**Published:** 2023-10-13

**Authors:** Ryaan EL-Andari, Rami Zibdawi, Paula Holinski, John Koller, Chloe Joynt, Nee Khoo, Laurance Lequier, Hamdy El-Hakim, Mohammed Al Aklabi

**Affiliations:** a Division of Cardiac Surgery, University of Alberta, Edmonton, Alberta, Canada; b Faculty of Medicine and Dentistry, University of Alberta, Edmonton, Alberta, Canada; c Department of Anesthesiology and Pain Medicine, University of Alberta, Edmonton, Alberta, Canada; d Division of Neonatology, Department of Pediatrics, University of Alberta, Edmonton, Alberta, Canada; e Division of Pediatric Cardiology, Department of Pediatrics, University of Alberta, Edmonton, Alberta, Canada; f Division of Pediatric Critical Care, Department of Pediatrics, University of Alberta, Edmonton, Alberta, Canada; g Division of Otolaryngology—Head and Neck Surgery, Department of Surgery, University of Alberta, Edmonton, Alberta, Canada; h Division of Congenital Cardiac Surgery, University of Alberta, Edmonton, Alberta, Canada

## Abstract

**Background:**

Congenital malformations of the trachea are rare but often life-threatening. Limited data have been published on the outcomes of tracheal reconstruction for congenital tracheal stenosis. We sought to describe the outcomes of patients undergoing tracheal reconstruction over 10 years at our centre.

**Methods:**

All paediatric patients who underwent long-segment tracheal or bronchial reconstruction from January 1, 2012, to August 31, 2022, were included. The primary outcome was mortality, and secondary outcomes included reoperation and postoperative morbidity. Patients were followed up to 10 years.

**Results:**

Thirty-three patients with ages ranging from 1 day to 12 years (mean 8.5 months) at the time of tracheoplasty or bronchoplasty were included, with 5 patients undergoing off-pump tracheal reconstruction. The most common preoperative comorbidities included patent ductus arteriosus (30.3%), atrial septal defect (27.3%), and prematurity (24.2%). There were no deaths postoperatively within the follow-up period. All patients experienced successful reconstruction with no patients requiring reoperation of the trachea. A total of 14 patients (42.4%) required postoperative balloon dilation, 3 (9.1%) required bronchial repair after tracheal repair, and 2 (6.1%) required bronchoscopic tracheal debridement.

**Conclusions:**

This single-centre retrospective study provides a large cohort of congenital tracheal reconstruction patients with a survival rate of 100%, experiencing no mortality during follow-up. The majority of patients had preoperative comorbidities and concomitant congenital cardiac defects. Although tracheal reconstruction continues to be complex with significant postoperative morbidity and mortality, the results of our single-centre study demonstrate the continual advancement of this field and the evolving improvement of postoperative outcomes for these patients.

## Résumé

**Contexte:**

Les malformations congénitales de la trachée sont rares, mais elles menacent souvent le pronostic vital. Peu de données ont été publiées sur les résultats cliniques de la reconstruction trachéale dans les cas de sténose trachéale congénitale. Notre objectif était de décrire les résultats obtenus par les patients ayant subi une reconstruction trachéale dans notre établissement sur une période de 10 ans.

**Méthodologie:**

Les données de tous les enfants ayant subi une reconstruction d’un long segment trachéal ou bronchique entre le 1^er^ janvier 2012 et le 31 août 2022 ont été incluses dans l’étude. Le critère principal d’évaluation était la mortalité et les critères secondaires d’évaluation incluaient la réintervention chirurgicale et la morbidité postopératoire. Les patients ont été suivis pendant 10 ans.

**Résultats:**

Trente-trois patients âgés de 1 jour à 12 ans (moyenne de 8,5 mois) au moment de la trachéoplastie ou de la bronchoplastie ont été inclus dans l’étude, dont 5 ayant subi une reconstruction trachéale sans circulation extracorporelle (*off-pump*). Avant l’intervention, les affections concomitantes les plus fréquentes étaient la persistance du canal artériel (30,3 %), la communication interatriale (27,3 %) et la prématurité (24,2 %). Aucun décès postopératoire n’est survenu au cours de la période de suivi. La reconstruction a été un succès chez tous les patients, et aucun d’entre eux n’a dû subir de nouvelle opération trachéale. Une dilatation postopératoire par ballon a été nécessaire chez 14 patients (42,4 %), 3 d’entre eux (9,1 %) ont subi une correction chirurgicale des bronches après la reconstruction trachéale, et 2 (6,1 %) ont subi un débridement trachéal par bronchoscopie.

**Conclusions:**

Notre étude rétrospective monocentrique dresse le portrait d’une vaste cohorte de patients ayant subi une reconstruction de la trachée en raison d’une malformation congénitale, parmi laquelle le taux de survie était de 100 %, sans aucun décès au cours de la période de suivi. Par ailleurs, la majorité des patients présentaient, avant l’intervention, des affections et des malformations cardiaques concomitantes. La reconstruction trachéale demeure malgré tout complexe et les taux de mortalité et de morbidité postopératoires restent importants, mais les résultats de notre étude monocentrique témoignent des progrès continuels dans ce domaine et de l’amélioration des résultats postopératoires pour ces patients.

**Figure 1:**
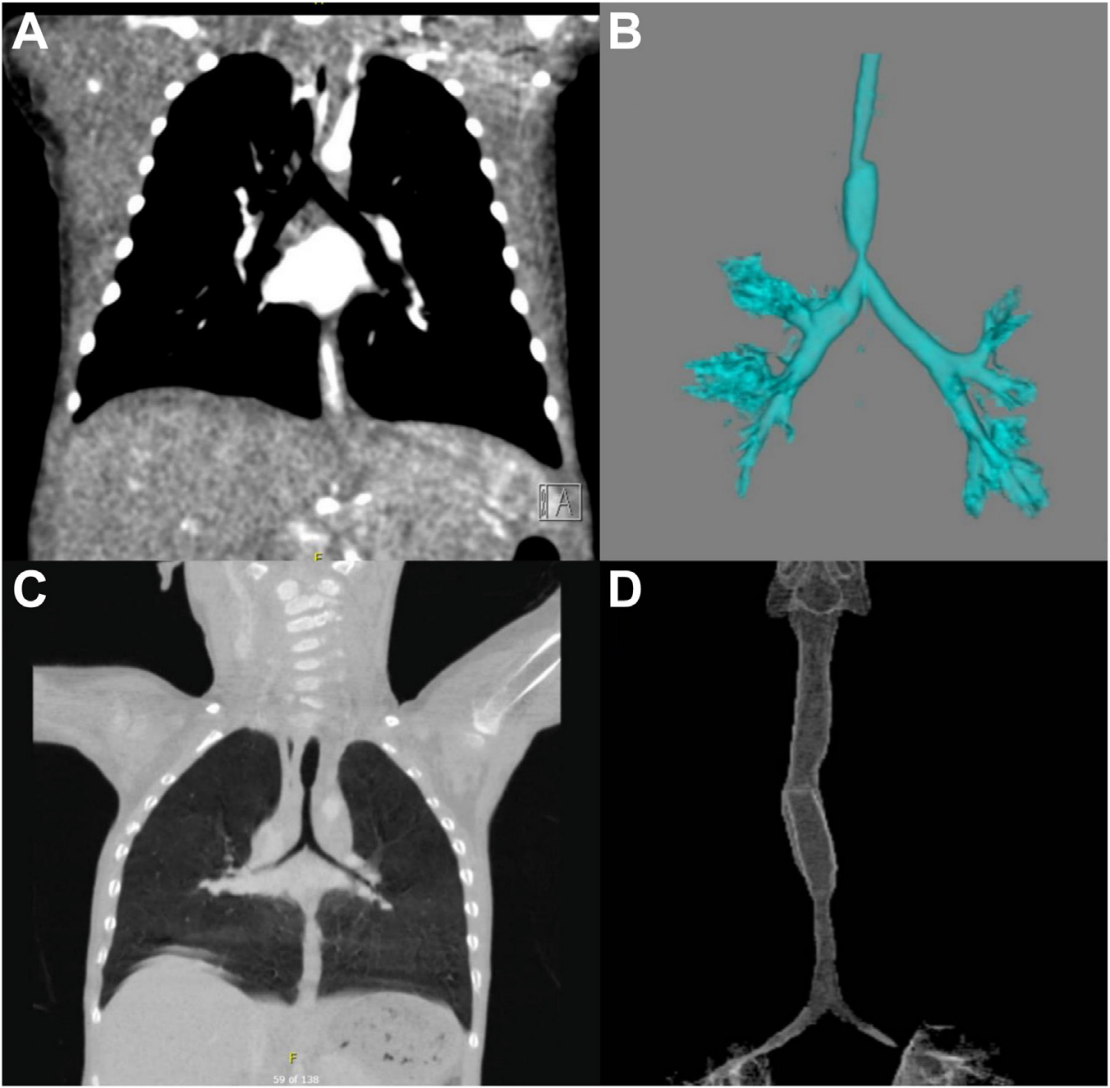
Preoperative computerized tomography scan of a 3-week-old patient showing focal tracheal stenosis narrowing to 1 mm immediately superior to the carina (**A**) and 3-dimensional reconstruction of the trachea (**B**), computerized tomography scan of a 28-month-old patient with long-segment tracheal narrowing from the level of the aortic arch to the carina, in keeping with complete tracheal rings (**C**) and 3-dimensional reconstruction of the trachea (**D**).

Congenital malformations of the trachea are rare but often life-threatening deformities that affect approximately 1 in 64,500 live births.^1^^,^^2^ In adults, the most common causes of tracheal stenosis are postintubation airway stenosis, tracheoesophageal fistula, trauma, and malignancy.^2^^,^^3^ Congenital tracheal stenosis (CTS), primary tracheomalacia (intrinsic weakness in the cartilaginous wall of the trachea), and secondary tracheomalacia (external compression by anomalous thoracic anatomy) are the most common causes in paediatric populations. CTS is often caused by an absence of the posterior membranous portion of the trachea being replaced by complete tracheal rings.^1^^,^^2^^,^^4^^,^^5^ CTS can affect any section or length of the trachea (Fig. 1), with long-segment tracheal stenosis generally defined as stenosis affecting >50% of the trachea.^1^^,^^6^ In paediatric populations, presentation most often includes symptoms of stridor and respiratory distress.^7^

Severe congenital malformations of the trachea often require surgical repair. Because of the nature of the surgical procedures, the use of cardiopulmonary bypass (CPB) or extracorporeal membrane oxygenation is often needed.^6^^,^^8^^,^^9^ The simplest tracheal reconstruction technique is an end-to-end anastomosis involving transecting the trachea, resecting the stenotic segment, and anastomosing the 2 ends. This technique is limited by its ability to address longer lesions. Tracheal stenosis requiring resection and advancement beyond 30% of the trachea’s original length is difficult to address with this technique and often requires further augmentation.^1^^,^^4^^,^^10^, ^11^, ^12^ Another option is a pericardial patch tracheoplasty. This involves creating a vertical incision in the stenotic segment of the trachea that is closed with a pericardial patch enlarging the tracheal segment.^1^^,^^6^^,^^8^^,^^10^^,^^13^ Although definitive surgical repair is the standard of care for these patients, interventional techniques such as tracheal stenting provide a palliative option when surgical intervention is contraindicated.^14^ The slide tracheoplasty, first described by Tsang et al.,^15^ involves tracheal transection performed in the midpoint of the stenosis. The trachea is dissected both superiorly and inferiorly. The 2 sections of the trachea are incised in mirror images of each, and the 2 mirror images are then brought together and anastomosed.^1^^,^^6^^,^^15^ The final option is a replacement of the trachea with a graft.^3^^,^^10^^,^^13^^,^^16^^,^^17^

Although surgical intervention has demonstrated significant benefits to these patients, surgical reconstruction is high risk. Mortality rates from tracheal reconstruction have been demonstrated to be between 0% and 50% in the literature, and postoperative morbidity may be as high as 40%.^1^^,^^2^^,^^4^^,^^8^, ^9^, ^10^, ^11^, ^12^^,^^18^, ^19^, ^20^, ^21^, ^22^, ^23^, ^24^, ^25^, ^26^, ^27^, ^28^, ^29^ Common complications after tracheal reconstruction include infection, intra- and postoperative bleeding, vocal cord paresis, tracheal obstruction, and growth of tracheal granulation tissue.^4^^,^^24^ Factors that contribute to the difficulty of this repair include complex anatomy, increased incidence of other congenital malformations, and infrequent case presentation.^1^, ^2^, ^3^, ^4^^,^^12^^,^^16^^,^^19^^,^^21^^,^^22^^,^^24^^,^^25^ Tracheal reconstruction is a relatively rare procedure with low volumes even at large referral centres, and thus, previous studies have been limited in their sample sizes. In recent years, larger cohorts of patients who underwent tracheoplasty have been reported, although morbidity and mortality continue to be significant.^26^, ^27^, ^28^, ^29^ Herein, we discuss the surgical approach used at our centre and the outcomes of 33 patients who have undergone tracheal reconstruction with a modified slide tracheoplasty or bronchoplasty. This cohort of tracheal and bronchial reconstruction patients is notable for being among the first to report 100% survival at long-term follow-up in a cohort of this size, with a subset of patients receiving tracheal reconstruction without the use of CPB.

## Materials and Methods

### Study cohort

Data were collected for all consecutive paediatric patients who had undergone tracheal reconstruction surgery from January 1, 2012, and February 28, 2022, at the Mazankowski Alberta Heart Institute and Stollery Children’s Hospital, a quaternary referral centre in Edmonton, Alberta. The slide tracheoplasty or bronchoplasty was the procedure of choice for all patients with long-segment tracheal or bronchial stenosis. The indications for surgery include the presence of significant tracheal stenosis, the presence of complete tracheal rings on endoscopy or computed tomography scan, and the presence of symptoms. Patients were reviewed by a multidisciplinary team including congenital cardiac surgery, otolaryngology, paediatric intensive care, and anaesthesia, among others before surgery. Exclusion criteria included patients >18 years of age. Thirty-three patients were included in this study with the majority presenting with congenital long-segment or bronchial tracheal stenosis and one notable case for foreign body aspiration for which the same surgical technique was used (Table 1). Presenting signs and symptoms varied from dyspnea, increased work of breathing, repeated respiratory tract infections, and acute respiratory distress, among others, after which tracheal pathology was confirmed on imaging. Postoperative outcomes and follow-up information were collected from postoperative assessments performed at the Stollery Children’s Hospital.

**Table 1:** Baseline characteristics of patients undergoing tracheal or bronchial reconstruction (N =33)

Baseline characteristics	Values
Average age at the time of surgery (IQR: Q1-Q3)	1.2 y (1.8-15.6 mo)
Weight at the time of surgery (IQR: Q1-Q3)	7.6 kg (3-7.9 kg)
Intubated preoperatively, n (%)	5 (15.2)
Tracheostomy preoperatively, n (%)	2 (6.1)
*Tracheal or bronchial pathology, n (%)*	
Complete tracheal rings/tracheal stenosis	26(78.8)
Tracheomalacia/bronchomalacia	6(18.2)
Bronchial stenosis	5(15.2)
Tracheoesophageal fistula	4(12.1)
Tracheal pouch	1(3.0)
Foreign body ingestion	1(3.0)
Tracheal bands	1(3.0)
*Preoperative comorbidities, n (%)*	
Pulmonary artery sling	12 (36.4)
Patent ductus arteriosus	10 (30.3)
Atrial septal defect	9 (27.3)
Prematurity	8 (24.2)
Ventricular septal defect	4 (12.1)
Trisomy 21	4 (12.1)
Pulmonary atresia	3 (9.1)
Cleft lip and/or palate	2 (6.1)
Esophageal atresia	2 (6.1)
Hypoplastic right ventricle	2 (6.1)
Right aortic arch	2 (6.1)
Subaortic stenosis	2 (6.1)
Vocal cord paralysis	2 (6.1)
Other comorbidities	1 (3.0)

Other comorbidities include (n = 1 patient each) aglossia, aortic aneurysm, asplenia, bicuspid aortic valve, choanal atresia, coarctation of the aorta, duodenal atresia, GERD, Goldenhar syndrome, hypoplastic left atrium, intracranial haemorrhage, laryngeal paralysis, micrognathia, neonatal entereocolitis, polyhydramnios, preoperative tracheostomy, previous cardiac surgery, pulmonary arterial hypertension, renal agenesis single umbilical artery, spinal stenosis, tetralogy of Fallot, upper abdominal wall haemangioma, VACTERLsequence, and valvular pathology.

GERD, gastroesophageal reflux disease; IQR, interquartile range.

### Surgical detail

**Figure 2:**
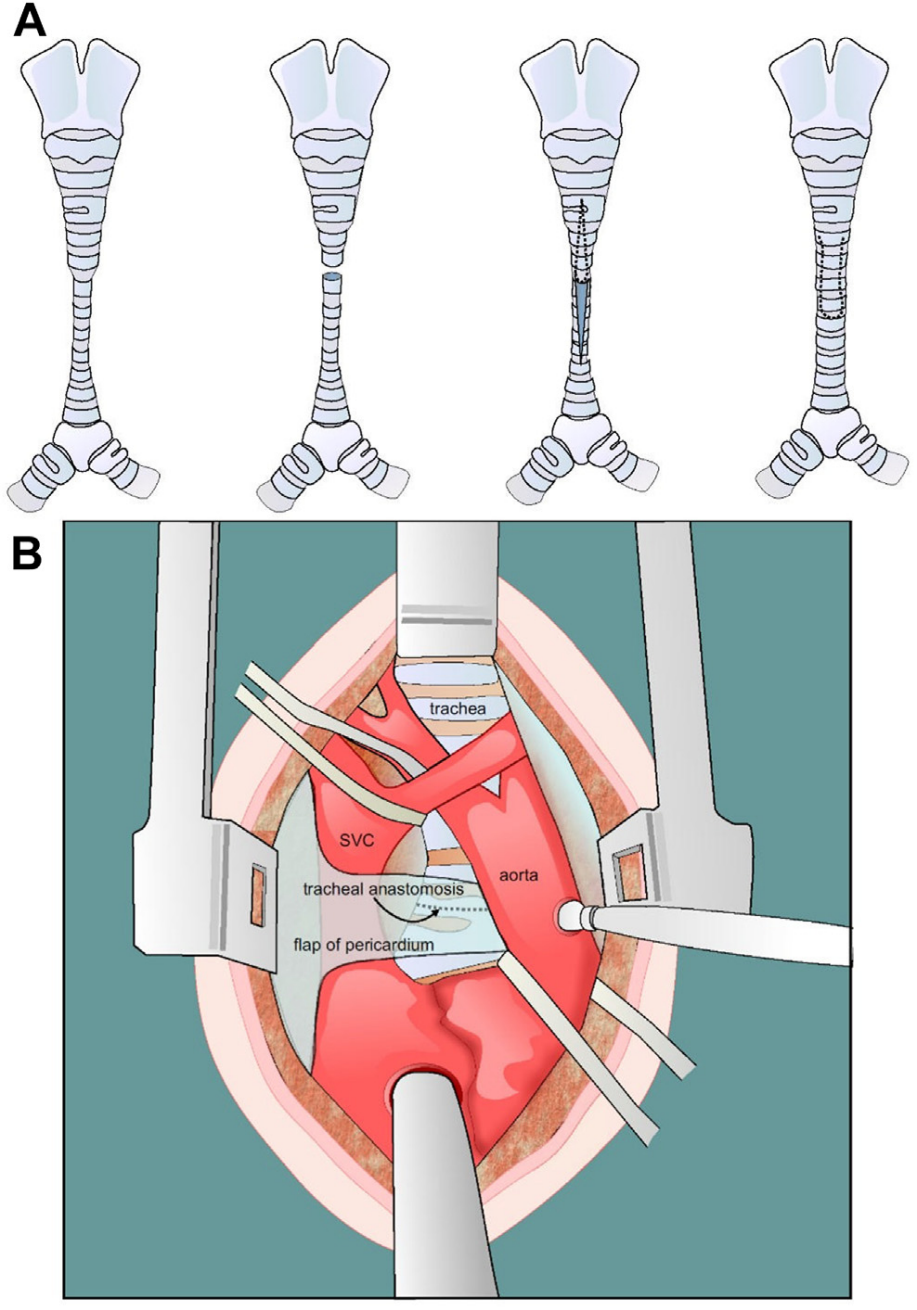
Image illustrating stenotic segment of the trachea repaired with a slide tracheoplasty (**A**) and placement of the pericardial flap over the superior vena cava and tracheal suture line (**B**). SVC, superior vena cava.

**Figure 3:**
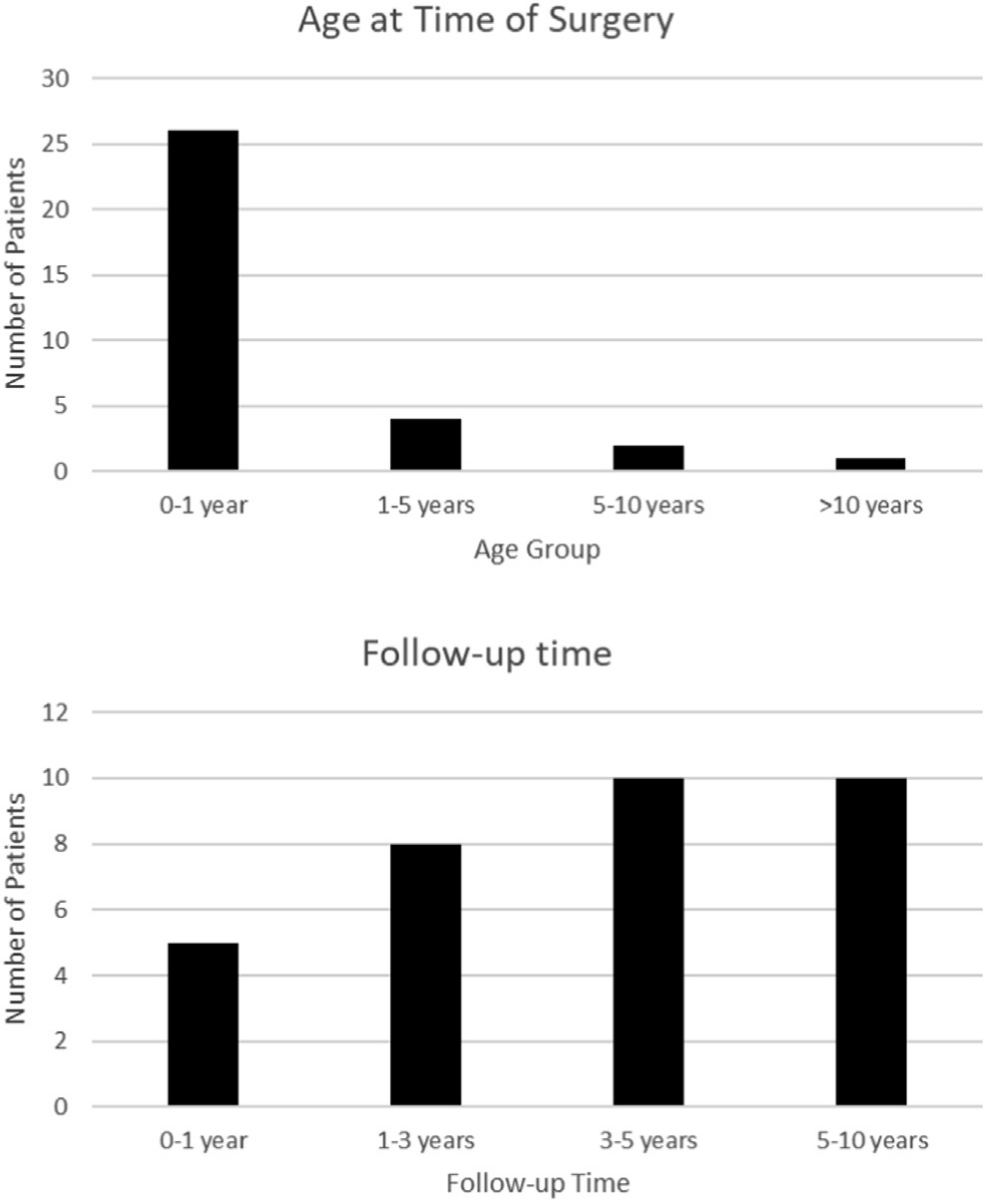
Bar graphs representing the number of patients based on age at the time of surgery and duration of follow-up.

The standard preoperative imaging was a computerized tomography scan for surgical planning, along with chest radiograph and echocardiography to identify patient anatomy and other concomitant conditions. Surgical technique varied slightly from case to case based on whether or not CPB was required, the patient’s comorbidities, and required concomitant surgeries. In general, after median sternotomy and institution of CPB (where applicable), slide tracheoplasty was performed by initially mobilizing the trachea proximally and distally towards the carina preserving important lateral blood supply. The trachea was then mobilized using bipolar cautery or diathermy very close to the trachea to protect the recurrent laryngeal nerve on each side. Bronchoscopy was used to identify the most stenosed portion of the trachea. At the most narrow point, the trachea was divided transversely. A longitudinal incision was then made on the anterior aspect of the lower end of the trachea towards the carina. On the upper segment of the trachea, another longitudinal incision was then made on the posterior aspect through the membranous portion for a similar length as the incision made in the lower trachea. Tracheoplasty was performed using between 5-0 and 7-0 polydioxanone sutures, depending on the patient size, running posteriorly and interrupted sutures at the anterior aspect towards the carina (Figs. 2 and 3).

When performing this anastomosis off pump, the endotracheal tube is pulled back proximally to the incision, and a sterile endotracheal tube is introduced into the field and advanced distally towards the carina and to allow ventilation while the posterior aspect of the anastomosis is performed. Once the posterior anastomosis is complete, the sterile endotracheal tube is removed from the field, and the previous endotracheal tube is advanced across the posterior aspect of the incision allowing for the completion of the anterior anastomosis. This technique allows for continual ventilation of the patient during the tracheal anastomosis without the need for CPB.

Intraoperative bronchoscopy was performed after anastomosis to inspect the trachea. In addition, a Valsalva maneuver is performed to check for anastomotic air leaks. In the case of slide bronchoplasty, the same technique described above is performed on the bronchus with longitudinal incisions made anteriorly and posteriorly, the 2 sides approximated and sutured in the same fashion as described for the tracheoplasty. A piece of autologous pericardium is mobilized with one side still attached and the other placed on top of the anastomosis between the trachea and aorta. Postoperatively at our centre, there is a preference for early extubation on postoperative day 1 or 2, and this was achieved for the majority of patients in this cohort. Postoperative follow-up includes imaging and bronchoscopy performed before discharge and as needed as an outpatient, in addition to follow-up with their primary care physician every 6 months.

### Outcomes

The primary outcome was mortality during the follow-up period with patients followed up to 10 years postoperatively. Secondary outcomes include recurrent or residual tracheal stenosis defined by clinically significant stenosis of the trachea after tracheoplasty requiring surgical reintervention, required reoperation and the postoperative complications of vocal cord paresis diagnosed on bronchoscopy, new prolonged tracheostomy >1 month required after surgery, laryngeal paresis, diaphragmatic paresis, acute respiratory distress syndrome, cardiac arrest, cerebrovascular accident, infection, mediastinitis, pericardial effusion, pleural effusion, and pneumothorax.

## Results

### Baseline demographics

**Figure 4:**
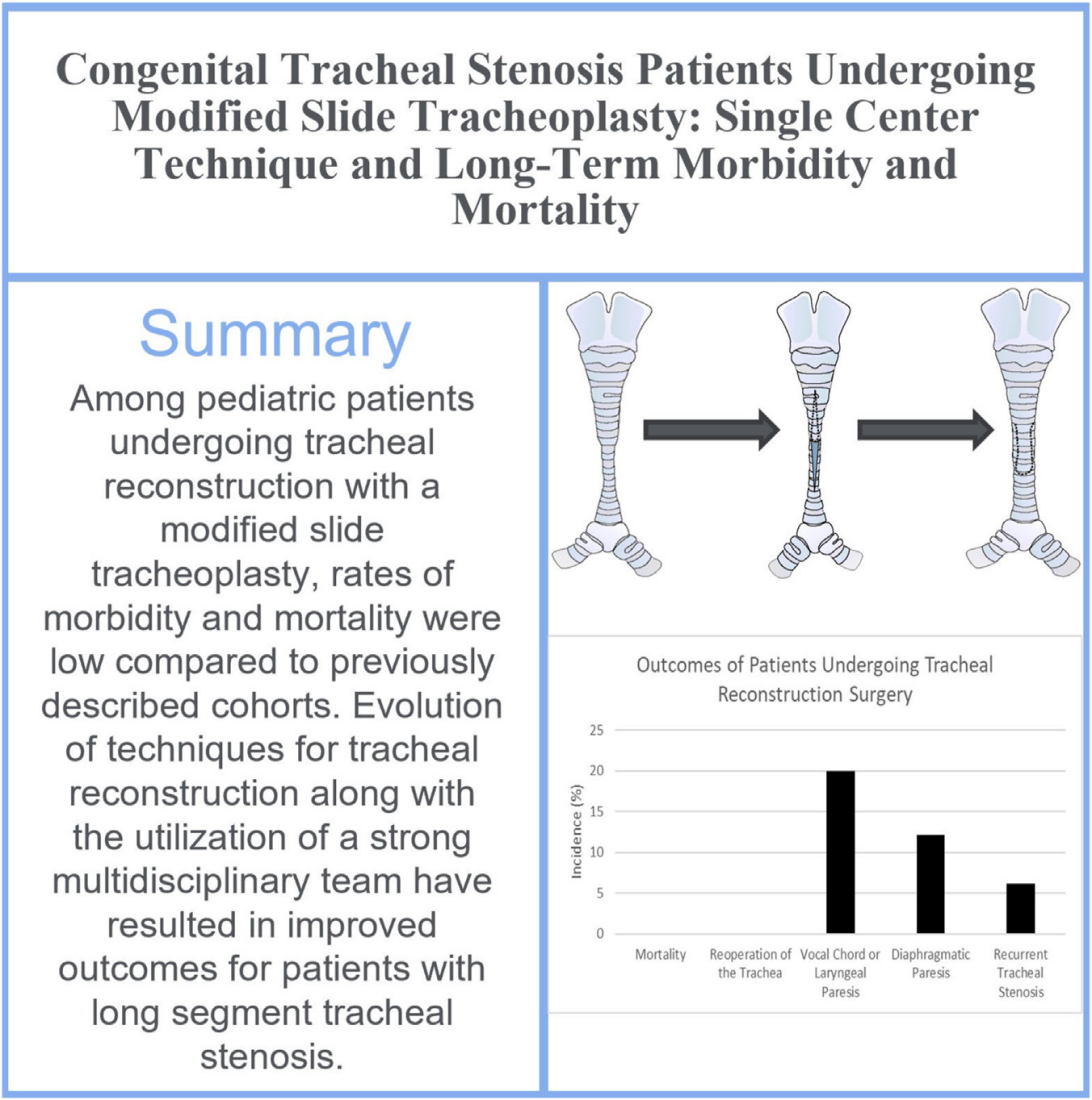
Central image: summary of a single-centre retrospective study of 33 patients who underwent reconstruction for congenital tracheal stenosis.

This study included 33 consecutive patients who had undergone tracheal or bronchial reconstruction surgery between January 1, 2012, and August 31, 2022. The age at the time of surgery ranged from 1 day to 12 years, with a mean age of 1.2 years (Fig. 4). Included in this cohort is the smallest patient reported to undergo successful tracheal reconstruction surgery weighing only 950 g at the time of surgery.^30^ Another case, previously described included a patient weighing 2 kg requiring extracorporeal membrane oxygenation for transportation from a referral centre over 1100 km away after which a successful tracheal reconstruction was performed.^31^ Tracheal pathology of the patients undergoing surgical intervention in this cohort included complete tracheal rings (78.8%), bronchial stenosis (15.2%), tracheoesophageal fistula (12.1%), tracheal pouch (3.0%), foreign body ingestion (3.0%), and tracheal bands (3.0%). The most common preoperative concomitant conditions included patent ductus arteriosus (30.3%), atrial septal defect (27.3%), prematurity (born at <37 weeks of gestation) (24.2%), ventricular septal defect (12.1%), trisomy 21 (12.1%), cleft lip and/or palate (6.1%), esophageal atresia (6.1%), hypoplastic right ventricle (6.1%), and subaortic stenosis (6.1%). At presentation for surgery, 5 patients were intubated and 2 had a tracheostomy (Table 1).

### Operative characteristics

Of the 33 patients who underwent tracheal or bronchial reconstruction surgery, 31 received a slide tracheoplasty and 11 underwent bronchial reconstruction. A total of 28 (84.4%) procedures were performed on CPB, whereas 5 (15.2%) were performed without CPB. The most common concomitant procedures for the included patients included pulmonary artery repair or reimplantation (n = 9) in order to reduce the risk of required reintervention of the pulmonary artery, atrial septal defect closure (9), ligation of the patent ductus arteriosus (8), aortopexy (3), right ventricular to pulmonary artery conduit (2), and ventricular septal defect repair (2) (Table 2). In 3 cases with a left pulmonary artery (LPA) sling, the LPA was not reimplanted. In these cases, the LPA was mobilized significantly and moved anteriorly to the trachea, and at the end of the operation, the LPA was found to have a suitable course without kinking or obstruction, and so they were left without intervention.

**Table 2:** Operative characteristics of patients undergoing tracheal and bronchial reconstruction (N = 33)

Operative characteristics	Values, n (%)
On cardiopulmonary bypass	28 (84.8)
Off cardiopulmonary bypass	5 (14.3)
Slide tracheoplasty	31 (93.9)
Pulmonary artery repair/reimplantation	9 (27.3)
Atrial septal defect closure	9 (27.3)
Ligation of patent ductus arteriosus	8 (24.2)
Bronchial reconstruction	5 (14.3)
Tracheoesophageal fistula repair	4 (12.1)
Aortopexy	3 (9.1)
Right ventricle-pulmonary artery conduit	2 (6.1)
Ventricular septal defect repair	2 (6.1)
Aortic repair	1 (3.0)
Primary tracheal repair	1 (3.0)
Pulmonary atresia repair	1 (3.0)
Tracheopexy	1 (3.0)

### Primary and secondary outcomes

The primary outcome of this study was mortality. No patients died during the initial procedure, hospital stay, or during the follow-up period. Patients were followed for a maximum of 10 years with a median follow-up of 4.1 years. No patients in this cohort required reoperation of the trachea during the follow-up period (Table 3).

**Table 3:** Postoperative characteristics of patients undergoing tracheal reconstruction (N = 33)

Characteristics	Values
*Postoperative outcomes*	
Average time to discharge	47.9 d
Hospital length of stay >30 days	18
Hospital length of stay >90 days	2
Mean intensive care unit length of stay	15.7 d
Intensive care unit length of stay >30 days	5
Average duration of postoperative intubation (range)	6.2 d (1 d to >1 y)
Average follow-up time (y)	4.1
Mortality during follow-up period, n (%)	0 (0)
Required tracheal reoperation, n (%)	0 (0)
*Bronchoscopic interventions, n (%)*	
Balloon dilation	14 (42.4)
Bronchoscopic debridement of the trachea	2 (6.1)
*Postoperative complication, n (%)*	
Vocal cord/laryngeal paresis	7 (20.0)
Required reintubation	5 (15.2)
Diaphragmatic paresis	4 (12.1)
Required prolonged CPAP	4 (12.1)
Residual or recurrent tracheal stenosis	2 (6.1)
New prolonged tracheostomy	1 (3.0)
Acute respiratory distress syndrome	1 (3.0)
Cardiac arrest	1 (3.0)
Cerebrovascular accident	1 (3.0)
Surgical site infection	1 (3.0)
Mediastinitis	1 (3.0)
Pericardial effusion	1(3.0)
Pleural effusion	1 (3.0)
Pneumothorax	1 (3.0)

CPAP, continuous positive airway pressure.

A total of 27 patients experience postoperative complications or required bronchoscopic interventions including vocal cord or laryngeal paralysis (7 patients, 20.0%), required reintubation (5 patients, 15.2%), diaphragmatic paresis (4 patients, 12.1%), required continuous positive airway pressure (4 patients, 12.1%), residual or recurrent tracheal stenosis (2 patients, 6.1%), new prolonged postoperative tracheostomy (1 patient, 3.0%), acute respiratory distress syndrome (1 patient, 3.0%), cardiac arrest (1 patient, 3.0%), cerebrovascular accident (1 patient, 3.0%), infection (1 patient, 3.0%), mediastinitis (1 patient, 3.0%), pericardial effusion (1 patient, 3.0%), pleural effusion (1 patient, 3.0%), and pneumothorax (1 patient, 3.0%). A total of 13 patients (37.1%) required postoperative balloon dilation and 2 (5.7%) required bronchoscopic debridement of the trachea. The average time spent intubated postoperatively was 6.2 days. Three patients required prolonged postoperative tracheostomy with 2 having a tracheostomy preoperatively and 1 requiring a new postoperative tracheostomy. Two patients required prolonged tracheostomy for difficulty weaning ventilation, and another for combined congenital disease and craniofacial abnormalities. Four other patients required postoperative intubation for >2 weeks. The indication for prolonged intubation was most often difficulty weaning ventilation with reasons including tracheo/broncheomalacia, diaphragmatic paresis, and atelectasis (Table 3).

## Discussion

Several interesting findings were identified in the analysis of this cohort. First, in contrast to the previous literature that has demonstrated high rates of morbidity and mortality in this patient population, there were no deaths during the follow-up period among the patients at our centre undergoing tracheal reconstruction. This was true despite the majority of the patients included having comorbidities and concomitant congenital defects such as congenital heart defects, esophageal atresia, Goldenhar syndrome, and VACTERL sequence, among others. Second, all patients experienced a successful reconstruction, and no patients required reoperation of the trachea during the follow-up period. Although our maximum duration of follow-up was 10 years, the mean follow-up time was 4.1 years with some patients having shorter durations of follow-up. The surgical techniques used were also notable with the inclusion of 5 cases of slide tracheoplasty performed without the use of CPB along with the addition of a mobilized pericardial patch used to prevent erosion and fistula formation between the trachea and aorta. Finally, the most common postoperative complications included vocal cord, laryngeal, and diaphragmatic paresis. Although these complications and others have been described in high rates within other cohorts, our cohort was at relatively low risk of developing such complications.

Tracheal reconstruction surgeries are highly complex surgical procedures and require specialized pre- and postoperative care, especially in the context of CTS. Various reconstruction approaches exist and are used based on pathology. Airway management is especially difficult in these patients, and there are often associated congenital cardiac, pulmonary, or abdominal malformations that require concurrent correction and contribute to the increased complexity of these cases.^1^^,^^2^ In addition, tracheal reconstruction surgeries are infrequent resulting in low volumes at individual centres and difficulty for surgeons in attaining significant experience with these cases. These combined factors have contributed to the significant mortality rates experienced by these patients, with mortality between 0% and 40% and morbidity as high as 50% during follow-up.^1^^,^^2^^,^^4^^,^^7^, ^8^, ^9^, ^10^, ^11^^,^^18^, ^19^, ^20^, ^21^, ^22^, ^23^, ^24^, ^25^, ^26^, ^27^, ^28^, ^29^ Although there have been previous publications describing the outcomes of tracheal reconstruction patients with sizeable cohorts including descriptions of cohorts from the Great Ormond Street Hospital, Cincinnati Children’s Hospital Medical Center, and those of Zhang et al.^26^ among others, our retrospective analysis of 33 patients over 10 years is among the first to demonstrate no mortality on long-term follow-up of a large cohort, including a subset of patients undergoing tracheal reconstruction without the use of CPB.^26^, ^27^, ^28^, ^29^^,^^32^ Our centre is a referral centre for all of western Canada resulting in a relatively high concentration of tracheal and bronchial reconstruction procedures performed at our centre with patients from a widespread geographic area.

The slide tracheoplasty has been the procedure of choice for long-segment tracheal stenosis at our centre for the past 10 years. The modified surgical approach used in this cohort includes avoiding the use of CPB and a partially interrupted anastomosis. Several criteria are required for safe off-pump tracheal reconstruction. The patient must not have a concomitant condition requiring repair with the assistance of CPB, there must be a sufficient length of the trachea at the distal aspect of the incision before the carina to allow for the placement of an endotracheal tube and inflation of the cuff for adequate ventilation, and there must be sufficient visualization with the endotracheal tube in place to perform the anastomosis. These criteria are required to facilitate safe off-pump tracheal reconstruction. This technique is also used in patients with extremely low weight who are not able to undergo CPB. In addition, our surgical technique uses an autologous pericardial patch that is harvested leaving one end attached to preserve blood flow. This patch is then placed at the distal anastomosis between the trachea and aorta where the interrupted sutures are placed, which prevents the interrupted suture knots from eroding into the aorta, prevents tracheoaortic fistula formation, accelerates healing, and helps encourage blood supply to the distal trachea. The results of this study demonstrate the success of the slide tracheoplasty procedure and the advances made in the realm of tracheal reconstruction surgery.

Several factors have contributed to the exceptional postoperative outcomes experienced at our centre including a modified surgical approach, relatively high volumes allowing for increased experience, and a strong multidisciplinary team including congenital cardiac surgery, otolaryngology, paediatric intensive care, anaesthesia, neonatology, nursing, respiratory therapy, and rehabilitation staff among others. The multidisciplinary team is imperative in safe surgical planning, intraoperative success, and management of postoperative complications. Close follow-up with a multidisciplinary team allows for early identification of complications and optimal management when required. A significant proportion of patients experienced postoperative morbidity including vocal cord or laryngeal paralysis, diaphragmatic paresis, tracheal air leak, tracheocutaneous fistula, and recurrent or residual tracheal stenosis. Similar outcomes have been reported in other studies.^1^^,^^11^^,^^18^^,^^20^ For example, in a study by Mainwaring et al.,^18^ of 27 patients who underwent tracheal surgery, 11% experienced postoperative sepsis, 7% experienced prolonged pleural effusions, and 4% experienced heart block or a tracheocutaenous fistula. Sidell et al.^20^ reported the outcomes of 52 patients who underwent primary or revision tracheal surgery. Patients most commonly experienced prolonged ventilation (23.1%-38.5%), infection (0%-7.7%), restenosis (3.8%-7.7%), and symptomatic deformities (3.8%-15.4%). Three patients in this study required a prolonged tracheostomy postoperatively, with 2 of these having a tracheostomy preoperatively. In all cases, tracheostomy was required for difficulty ventilating the patients with standard intubation. Among these patients, tracheostomy was recommended for up to 1 year after which the patient would be reassessed for extubation. One patient remains tracheostomy dependent due to several congenital conditions and craniofacial abnormalities resulting in difficulty with respiration. Although morbidity was reported in our study, the most common morbidities were diaphragmatic, laryngeal, or vocal cord paresis with other morbidities occurring infrequently in less than 5% of patients. Strategies have been described for the improvement of postoperative morbidity in this patient population. For example, intraoperative nerve mapping has been used with other surgical procedures in order to avoid intraoperative nerve injuries. One study compared the outcomes of adult patients undergoing tracheal surgery between groups where intraoperative nerve monitoring had been used vs those where it had not. There was no significant difference in rates of nerve injury between the groups.^33^ In recent years, the field of 3-dimensional printing for modelling and preoperative preparation in tracheal reconstruction surgery has emerged. Three-dimensional modelling allows for a more detailed understanding of the patient’s anatomy, allowing the surgeon to anticipate challenging aspects of the surgery, helping to plan cases, and avoid pitfalls. The models also allow for preoperative trials of various techniques allowing surgeons to select the approach that will work best for the individual patient.^34^ Continued efforts to improve planning in complex cases and avoid common pitfalls such as those described above will contribute to continually improving outcomes for this patient population.

The advanced complexity of patients with CTS and the surgical intervention required for correction will continue to pose challenges in the management of this condition. The localization of these procedures to few centres allows for increased volumes and experience; in addition, a strong team specialized in tracheal reconstruction will provide improved conditions to achieve optimal outcomes. Furthermore, the publication of postoperative outcomes and surgical techniques will continually evolve the knowledge surrounding tracheal reconstruction surgery allowing for collective growth in the surgical approach and interventions for CTS and improvement in the outcomes of these patients. Moving forward, the publication of combined cohorts from multiple centres or the creation of a multicentre registry will allow for the analysis of larger populations of paediatric patients undergoing tracheal reconstruction and may allow for in-depth analyses of factors predictive of outcomes.

### Limitations

The retrospective nature of this study is the primary limitation. In addition, this centre has a wide catchment area spanning multiple provinces. This results in some outcomes potentially not being captured by this database. Measures of quality of life such as dyspnea score, phonation, and functional capacity were not captured in this dataset, although they are important for future studies to consider. Finally, although one of the larger cohorts of tracheoplasty patients, the sample size is still small and diverse limiting our ability to identify predictors of outcomes.

## Conclusions

In recent years, larger cohorts of tracheal reconstruction patients have been described in the literature, although morbidity and mortality continue to be significant. The results from our single-centre retrospective study provide one of the largest cohorts in the literature to experience no mortality at long-term follow-up and include a subset of patients who received tracheal reconstruction without the use of CPB. Similar to the previous literature, the majority of our cohort had preoperative comorbidities or concomitant congenital defects. Although postoperative complications included vocal cord and laryngeal paralysis, the incidence was relatively low in our cohort compared with previously studied cohorts. Essential to the success of a tracheal reconstruction programme is a strong multidisciplinary team. This team is imperative for preoperative surgical planning, intraoperative success, and postoperative management. Although technically successful, complications during the postoperative course are not uncommon due to the complex nature of these patients. The ability to manage postoperative complications is essential in the care of patients with tracheal stenosis. Although tracheal reconstruction continues to be complex with significant postoperative morbidity and mortality, the results of our single-centre study demonstrate the continual advancement of this field of surgery and the improving postoperative outcomes of these patients. Future studies using larger cohorts of patients are required for a better understanding of predictive factors for postoperative outcomes and quality-of-life metrics in this patient population.

## References

[1] Arcieri L., Pak V., Poli V. (2018). Tracheal surgery in children: outcome of a 12-year survey. Interact Cardiovasc Thorac Surg.

[2] Hewitt R.J., Butler C.R., Maughan E.F., Elliott M.J. (2016). Congenital tracheobronchial stenosis. Semin Pediatr Surg.

[3] Etienne H., Fabre D., Gomez Caro A. (2018). Tracheal replacement. Eur Resp J.

[4] Mcmahon C.J., Ayoubi K., Mehanna R. (2019). Outcome of congenital tracheal stenosis in children over two decades in a national cardiothoracic surgical unit. Cardiol Young.

[5] Wright C.D., Graham B.B., Grillo H.C., Wain J.C., Mathisen D.J. (2002). Pediatric tracheal surgery. Ann Thorac Surg.

[6] Sengupta A., Murthy R.A. (2020). Congenital tracheal stenosis & associated cardiac anomalies: operative management & techniques. J Thorac Dis.

[7] Manning P.B. (2017). Tracheal reconstruction in the pediatric population: how I teach it. Ann Thorac Surg.

[8] Hazekamp M.G., Koolbergen D.R., Kersten J. (2009). Pediatric tracheal reconstruction with pericardial patch and strips of autologous cartilage. Eur J Cardiothorac Surg.

[9] Hasegawa T., Oshima Y., Matsuhisa H. (2016). Clinical equivalency of cardiopulmonary bypass and extracorporeal membrane oxygenation support for pediatric tracheal reconstruction. Pediatr Surg Int.

[10] Backer C.L., Mavroudis C., Gerber M.E., Holinger L.D. (2001). Tracheal surgery in children: an 18-year review of four techniques. Eur J Cardiothorac Surg.

[11] Chen S.-J., Wu E.-T., Wang C.-C. (2019). Excessive tracheal length in patients with congenital tracheal stenosis. Ann Thorac Surg.

[12] Morita K., Maeda K., Yabe K., Oshima Y. (2017). Management of congenital tracheal stenosis in the neonatal period. Pediatr Surg Int.

[13] den Hondt M., Vranckx J. (2017). Reconstruction of defects of the trachea. J Mater Sci Mater Med.

[14] Freitag L., Darwiche K. (2014). Endoscopic treatment of tracheal stenosis. Thorac Surg Clin.

[15] Tsang V., Murday A., Gillbe C., Goldstraw P. (1989). Slide tracheoplasty for congenital funnel-shaped tracheal stenosis. Ann Thorac Surg.

[16] Delaere P. (2012). Tracheal transplantation. Curr Opin Pulm Med.

[17] Abouarab A.A., Elsayed H.H., Elkhayat H. (2017). Current solutions for long-segment tracheal reconstruction. Ann Thorac Cardiovasc Surg.

[18] Mainwaring R.D., Shillingford M., Davies R. (2012). Surgical reconstruction of tracheal stenosis in conjunction with congenital heart defects. Ann Thorac Surg.

[19] Morita K., Yokoi A., Fukuzawa H. (2016). Surgical intervention strategies for congenital tracheal stenosis associated with a tracheal bronchus based on the location of stenosis. Pediatr Surg Int.

[20] Sidell D.R., Hart C.K., Tabangin M.E. (2018). Revision thoracic slide tracheoplasty: outcomes following unsuccessful tracheal reconstruction. Laryngoscope.

[21] Okamoto T., Nishijima E., Maruo A. (2009). Congenital tracheal stenosis: the prognostic significance of associated cardiovascular anomalies and the optimal timing of surgical treatment. J Pediatr Surg.

[22] Usui Y., Ono S., Baba K., Tsuji Y. (2018). Pitfalls in the management of congenital tracheal stenosis: is conservative management feasible?. Pediatr Surg Int.

[23] Vu H.V., Huynh Q.K., Nguyen V.D.Q. (2019). Surgical reconstruction for congenital tracheal malformation and pulmonary artery sling. J Cardiothorac Surg.

[24] Wang S., Zhang H., Zhu L. (2016). Surgical management of congenital tracheal stenosis associated with tracheal bronchus and congenital heart disease. Eur J Cardiothorac Surg.

[25] Yokoi A., Hasegawa T., Oshima Y. (2018). Clinical outcomes after tracheoplasty in patients with congenital tracheal stenosis in 1997-2014. J Pediatr Surg.

[26] Zhang H., Wang S., Lu Z. (2017). Slide tracheoplasty in 81 children: improved outcomes with modified surgical technique and optimal surgical age. Medicine (Baltimore).

[27] Stephens E.H., Eltayeb O., Mongé M.C. (2020). Pediatric tracheal surgery: a 25-year review of slide tracheoplasty and tracheal resection. Ann Thorac Surg.

[28] Beeman A., Ramaswamy M., Chippington S. (2022). Risk stratification of slide tracheoplasty for pediatric airway stenosis. Ann Thorac Surg.

[29] Manning P.B., Rutter M.J., Lisec A., Gupta R., Marino B.S. (2011). One slide fits all: the versatility of slide tracheoplasty with cardiopulmonary bypass support for airway reconstruction in children. J Thorac Cardiovasc Surg.

[30] Zibdawi R., El-Andari R., Noga M. (2022). Tracheal reconstruction for congenital tracheal stenosis: a 950-gram neonate. Ann Thorac Surg.

[31] Martin B.-J., Holinski P., Noga M., El-Hakim H., Al Aklabi M. (2019). Neonatal tracheal and intracardiac repair in a high-risk premature infant requiring preoperative ECMO transport. World J Pediatr Congenit Heart Surg.

[32] Bajaj Y., Cochrane L.A., Jephson C.G. (2012). Laryngotracheal reconstruction and cricotracheal resection in children: recent experience at Great Ormond Street Hospital. Int J Pediatr Otorhinolaryngol.

[33] Kadakia S., Mourad M., Badhey A. (2017). The role of intraoperative nerve monitoring in tracheal surgery: 20-year experience with 110 cases. Pediatr Surg Int.

[34] Shimojima N., Shimotakahara A., Tomita H. (2022). Simulated slide tracheoplasty for congenital tracheal stenosis using three-dimensional printed models. Pediatr Surg Int.

